# Traditional Medicine in a Global Environment

**DOI:** 10.1155/2014/326895

**Published:** 2014-04-27

**Authors:** Rainer W. Bussmann, Wendy Applequist, Narel Paniagua-Zambrana

**Affiliations:** ^1^WLBC, Missouri Botanical Garden, P.O. Box 299, St. Louis, MO 63166-0299, USA; ^2^Herbario Nacional de Bolivia, La Paz, Bolivia

Traditional medicine, both codified (e.g., Chinese medicine, Ayurveda, and Unami) and noncodified, has become a global movement with rapidly growing economic importance. In many Asian countries traditional medicine is widely used, even though Western medicine is often readily available. The number of visits to providers of traditional medicine in USA now exceeds by far the number of visits to primary care physicians. Many medicinal plant species are easily available in online trade, often without correct scientific identification and with possible contamination, which creates large safety concerns. In developing countries, uncodified traditional medicine is often the only accessible and affordable treatment available.

The globalization of traditional remedies, in particular from noncodified pharmacopoeia, leaves many questions unanswered: does the use of traditional medicine reflect major health issues? Some plants may have beneficial properties, while others can cause adverse reactions. Even when the herbal ingredients themselves have proven benefits and no known safety concerns, some of the administration methods may be harmful. Importantly, how can safety concerns associated with traditional medicines and practices be identified, monitored, and communicated to users and other stakeholders, and how can the safety and sustainability of the global supply of medicinals be ensured?

This first special issue on traditional medicine in a global environment contains 6 manuscripts covering different aspects of traditional medicine in a global setting.

Of these 6 manuscripts, two address use and conservation issues of traditional medicine in Nepal and Northern India, two address the evaluation of the biological activities of medicinal plants and their efficacy in South Africa and China, one looks at potentially problematic compounds in one of the most widely sold supplements, and finally one explores the changes of traditional medicine use in Northern Peru during more than a decade of research.

We hope that this collection of papers in this special issue will give our readers valuable insights into diverse areas of the subject.

*Rainer W. Bussmann* *Rainer W. Bussmann* *Wendy Applequist* *Wendy Applequist* *Narel Paniagua-Zambrana* *Narel Paniagua-Zambrana*

